# Redox nanoparticles: synthesis, properties and perspectives of use for treatment of neurodegenerative diseases

**DOI:** 10.1186/s12951-018-0412-8

**Published:** 2018-11-03

**Authors:** Izabela Sadowska-Bartosz, Grzegorz Bartosz

**Affiliations:** 1 Department of Analytical Biochemistry, Faculty of Biology and Agriculture, University of Rzeszow, Zelwerowicza Street 4, 35-601 Rzeszow, Poland; 2 Department of Molecular Biophysics, Faculty of Biology and Environmental Protection, University of Lodz, Pomorska Street 141/143, 90-236 Lodz, Poland

**Keywords:** Oxidative/nitrative stress, Protein aggregation, Redox nanoparticles, Alzheimer’s disease, Parkinson’s disease

## Abstract

Oxidative stress (OS) and nitrative stress (NS) accompany many diseases, including Alzheimer’s disease (AD) and Parkinson’s disease (PD). Antioxidants have been proposed to counteract OS/NS in these diseases. Nevertheless, the effects of antioxidants are limited and new, more efficient antioxidants are searched for. Redox-active nanoparticles (RNPs), containing antioxidants create a new therapeutical perspective. This review examines the recent literature describing synthesis and potential applications of cerium oxide RNPs, boron cluster-containing and silica containing RNPs, Gd_3_N_@_C_80_ encapsulated RNPs, and concentrates on nitroxide-containing RNPs. Nitroxides are promising antioxidants, preventing inter alia glycation and nitration, but their application poses several problems. It can be expected that application of RNPs containing covalently bound nitroxides, showing low toxicity and able to penetrate the blood–brain barrier will be more efficient in the treatment of neurodegenerative disease, in particular AD and PD basing on their effects in cellular and animal models of neurodegenerative diseases.

## Introduction

Age-related diseases such as Alzheimer’s disease (AD) and Parkinson’s disease (PD) constitute a considerable socioeconomic burden for contemporary societies. As human mean lifespan increases, growing incidence of these diseases has features of a pandemic. AD is one of the main causes of insufficiency and need of care by other persons, which significantly deteriorates the quality of life of both the affected persons and their caregivers. It should be emphasized that current AD and PD treatments alleviate only the symptoms of the disease without halting its progress. Oxidative stress (prooxidative disturbance in the equilibrium between prooxidants and antioxidants; OS) is observed in the course of many diseases and plays a role in the etiopathogenesis of some of them. Neurodegenerative diseases, including AD and PD are characterized by increased levels of protein nitration, which is mainly the result of the reactions of peroxynitrite (ONOO^−^) primarily with residues of such amino acids as tyrosine, phenylalanine and histidine [1–3]. Peroxynitrite is formed in vivo under conditions of OS/NS, in the diffusion-controlled reaction between nitric oxide (NO^•^) and superoxide radical anion (O_2_^•−^). Production of NO^•^ and O_2_^•−^ intensifies as a result of immune response, inter alia, by macrophages. Peroxynitrite has multiple actions on various molecules, especially proteins [4], including one- and two-electron oxidations, nitration (attachment of NO_2_) and nitrosylation (attachment of NO).

It is generally assumed that increased level of nitration, evidencing enhanced ONOO^−^ production, is a universal marker of inflammation; it was also found in such pathologies as AD, PD, amyotrophic lateral sclerosis, diabetes, rheumatoid arthritis, lupus, atherosclerosis, hypertension, as well as liver and cardiovascular diseases [5].

Antioxidants have been proposed to counteract the development of neurodegenerative diseases. However, the effects of antioxidant intervention, including increased consumption of dietary antioxidants, are generally modest in the best case [6, 7]. These findings suggest that new antioxidant compounds, of increased efficacy, should be searched for. Synthetic antioxidants may be more efficient than naturally occurring ones. Apart from the antioxidant function, synthetic compounds have other beneficial actions; e.g. they can interfere with protein aggregate formation or inhibit undesired enzymatic activities. Free nitroxide radicals (nitroxides) are promising in this respect. They are many-faceted antioxidants, which have pseudo-superoxide dismutase (SOD) activity, inhibit Fenton chemistry by the ability to oxidize transition metal ions, terminate radical chain reactions by radical recombination and accept electrons from the mitochondrial electron transport chain. They react also with protein tyrosyl and tryptophanyl radicals [8].

Nitroxides have been demonstrated to inhibit oxidation and, especially, nitration by reacting with ONOO^−^ and/or its decomposition products. Our comparative study of 11 nitroxides of various structure demonstrated structure-dependent differences in their efficiency, suggesting the possibility of choice and design of optimal compounds. Nitroxides were more effective in preventing nitration than oxidation reactions. Concentrations corresponding to IC_50_ for prevention of nitration of model compounds for most nitroxides were lower than 100 nM, which, in combination with the lack of changes of ESR signal of the nitroxides, speaks for catalytic decomposition of ONOO^−^ or its decomposition products by these compounds. No prooxidant effect of nitroxides was seen in prevention of dihydrorhodamine 123 (DHR123) oxidation induced by 3-morpholino-sydnonimine (SIN-1, OONO^−^ donor). Interestingly, nitroxides showed a concentration window for preventing DHR123 oxidation by ONOO^−^, exerting a prooxidant effect at both high and low concentrations. Nitroxides turned out to be much more effective in protecting human serum albumin (HSA) against nitration of tyrosine residues than against oxidation of thiol groups. Most nitroxides, except for the most hydrophobic ones (especially 4-nonylamido-TEMPO and 3-carbamoyl-dehydro-PROXYL) protected cells from the cytotoxic action of SIN-1 and SIN-1-induced protein nitration [9]. TEMPO, as well as an *N*-arylnitroxide and an *N*, *N*-diarylnitroxide, react with alkylperoxyl radicals, the propagating species in lipid peroxidation. They are significantly more reactive than Vitamin E, the most efficient natural antioxidant trapping peroxyl radicals. Nitroxides reduce peroxyl radicals by electron transfer with rate constants of 10^6^–10^7^ M^−1^ s^−1^. This reaction is catalytic; NADPH in the aqueous phase can be used as a hydride donor to promote nitroxide recycling. In cell culture, nitroxides are potent inhibitors of ferroptosis [10]. We found that nitroxides are glycation inhibitors: 2,2,6,6-tetramethylpiperidin-1-yl-oxyl (TEMPO), 4-carboxy-TEMPO and 4-hydroxy-TEMPO offered significant protection against glycoxidation induced by glucose and fructose, while 3-carbamoyl-PROXYL enhanced glycoxidation [11].

Nitroxides are not toxic to breast cancer MCF-7 cells (human breast adenocarcinoma cell line) at concentrations of up to 500 µM, with the exception of 4-cyano-TEMPO (significant reduction of survival at a concentration of 500 µM) and 4-nonylamido-TEMPO (significant reduction of survival in the concentration range of 250–500 µM) after 72-h incubation in the presence of these compounds. Preincubation of cells with nitroxides at a concentration of 100 µM enhanced survival of MCF-7 cells treated subsequently with 100 µM SIN-1. These compounds reduced also the level of intracellular protein nitration induced by SIN-1 [9]. Nitroxides have been shown to have antitumor activity and, consequently, they prolonged the lifespan of mice with an increased tendency for malignancy [12, 13]. It is important that nitroxides can be administered in vivo chronically. In fact, long-term administration of 4-hydroxy-TEMPO increased latency to tumorigenesis and doubled the lifespan of Atm-deficient mice and p53-deficient mice [13]. Nevertheless, it must be noted that low-molecular-weight (LMW) nitroxides pose several problems such as nonspecific dispersion in normal tissues, and preferential renal clearance resulting in short life time in vivo.

*Covalent attachment of nitroxides to nanoparticles (NPs) of optimal structure can be a solution of this problem, increase the efficacy of nitroxide action and allow for selective delivery*. Nanoparticles are natural or synthetic particles whose size is within the range of 1–100 nm. Nanoparticles are widely used in nanomedicine, playing an important role as diagnostic tool and being also applied for treatment, monitoring and control of many diseases, including neurodegenerative disorders. Many studies are focused on development of new drug delivery systems, which are necessary to increase the therapeutic effectiveness of NPs [14, 15]. Nowadays there are many approaches in drug delivery to the central nervous system (CNS). The most common strategies are based on polymeric NPs and lipid-based NPs; inorganic NPs (especially iron oxide and quantum dots) are also used. Neurotoxicity of NPs and their effect on neurons, cellular components and the blood–brain barrier (BBB) are equally important [16]. In order to prevent or at least limit adverse effects to normal organs, tissues and cells, disturbance of normal redox reaction in healthy tissues must be prevented. It can be achieved by optimal design and targeting of NPs.

In this review we concentrate on advantages of biocompatible and colloidally stable NPs, in which nitroxide radicals are covalently conjugated to a polymer structure (nitroxide-based redox nanoparticles, NRNPs). These NPs effectively scavenge reactive oxygen species (ROS) and reactive nitrogen species (RNS) with a characteristically prolonged bioavailability and tissue-residence time much longer than that of conventional LMW antioxidants. The confinement of the nitroxide radicals in the polymer core prevents their rapid metabolism and excretion out of the blood circulation. The nano-sized formulation limits internalization of NRNPs in healthy cells, thereby preserving the normal redox status of these cells.

### Structure of redox nanoparticles and synthesis methods

Nanoparticles are generally composed of three layers: (a) the surface layer, which may be functionalized with a variety of small molecules, surfactants, metal ions and polymers, (b) the shell layer, which is chemically different material from the core, and (c) the core, which is the central portion of the NP and usually is referred to as the NP itself [17, 18]. There are several methods for producing NPs, including gas condensation, attrition, chemical precipitation, ion implantation, pyrolysis and hydrothermal synthesis.

Nowadays many types of NPs are used. This review focuses on redox nanoparticles (RNPs), in particular NRNPs. Redox nanoparticles are biocompatible NPs that can scavenge ROS with high efficiency [19]. Redox nanoparticles are not internalized in normal cells by the intact cell membrane, so almost no cellular dysfunction is observed in normal cells after their administration [20]. Moreover, some RNPs (NRNP^N^, see below) disintegrate under acidic conditions due to the protonation of amino groups, because they possess amine linkages in the core. Accordingly, NRNP^N^s disintegrate into individual polymers within the stomach and can be absorbed as dissociated polymer into the bloodstream across the intestinal epithelium [21]. Most nanomedicines are not used as orally administered drugs for systemic diseases, since NPs of the size of 10–100 nm are not absorbed via the gastrointestinal tract. Redox polymers are absorbed by the blood following oral administration of RNPs, thereby rendering them an ideal medication for chronic diseases. Nanoparticle formulation can be drunk easily due to its low viscosity. This advantage is practically very important, considering the long-term treatment of chronic diseases [22]. Owing to large surface area-to-volume ratio of nanomaterials, higher electron densities can be found on the outer surface, resulting in increased catalytic activity [23]. For instance, metal oxide nanomaterials can potentially be involved in effective ROS scavenging and/or deoxygenating reactions [22, 24]. In recent years, an increasing number of studies have emphasized that nanomaterials can mimic the properties of antioxidant enzymes (nanomaterial-based artificial enzymes, so-called nanozymes), to inhibit apoptosis and improve the cell survival [25].

A few examples of RNPs are presented below.

### Cerium oxide (CeO_2_) nanoparticles (CNPs)

**Fig. 1:**
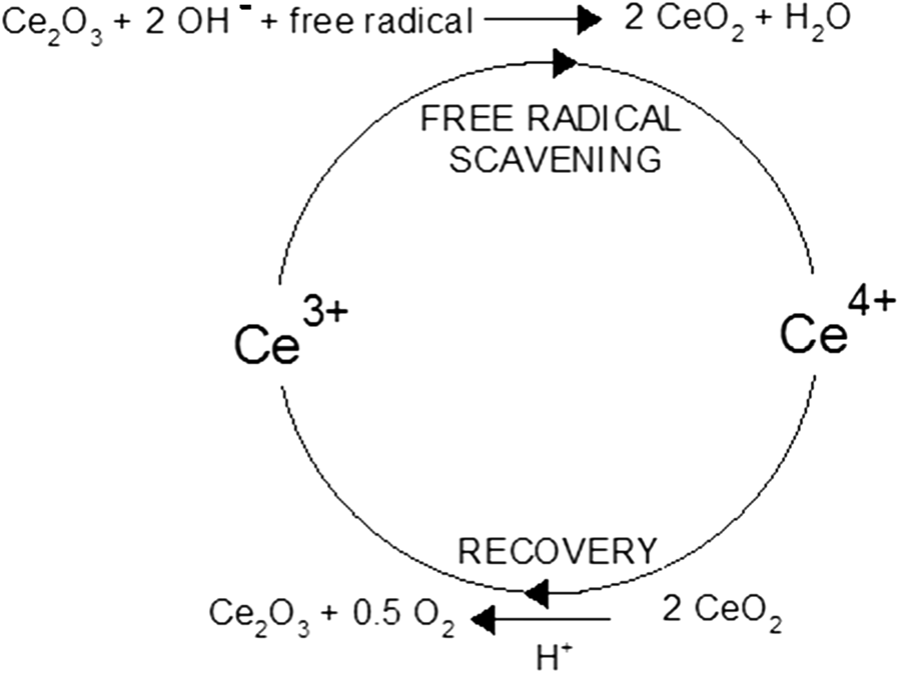
Autocatalytic behavior of cerium oxide nanoparticles (CNPs) in water, resulting in generation of free radicals

**Fig. 2:**
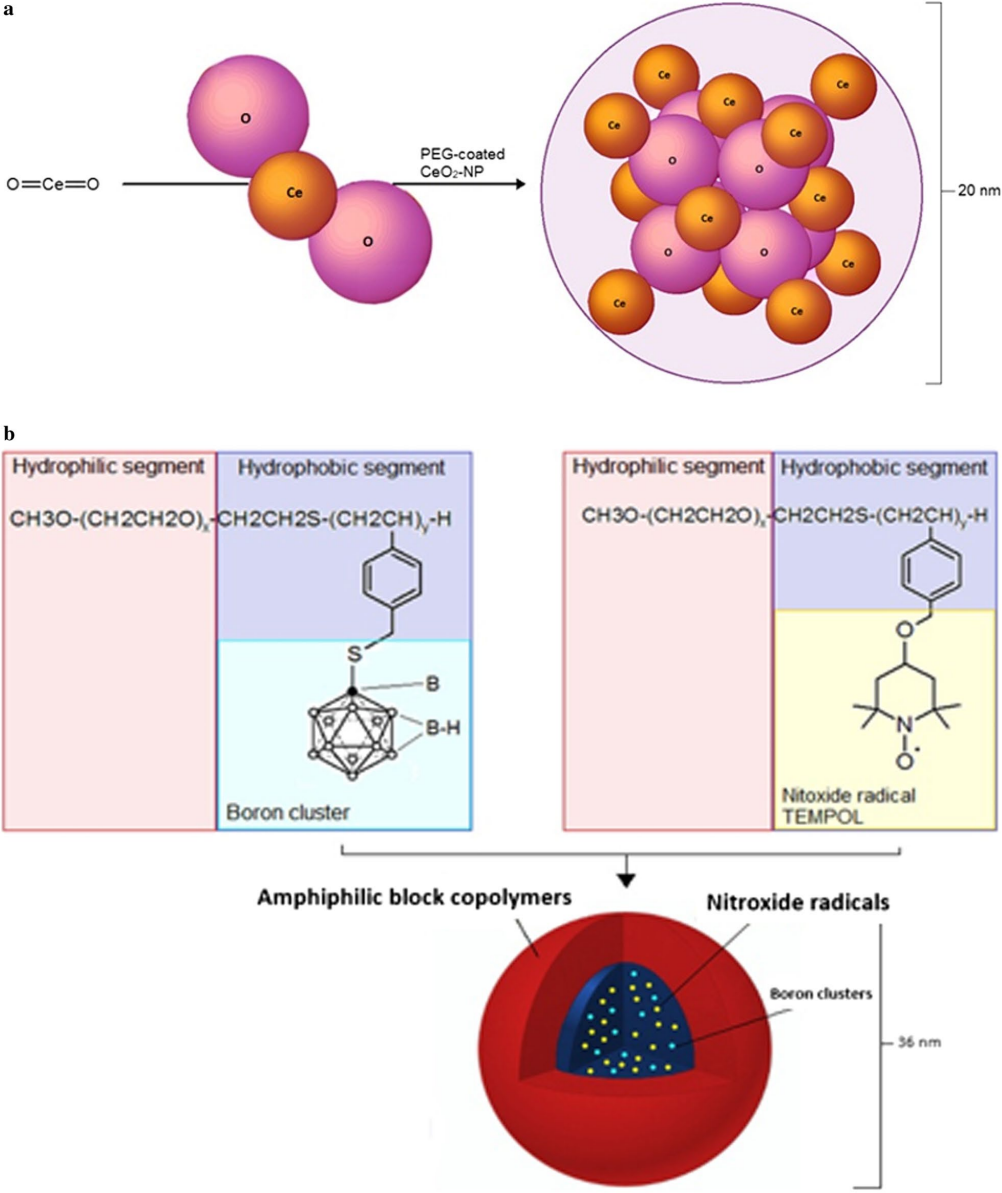
Structure of **a** cerium oxide nanoparticle (CNP) and **b** boron cluster-containing redox nanoparticle (B-RNP)

Cerium dioxide (ceria/CeO_2_), popularly known as nanoceria, has been reported to be the most stable oxide of the cerium. Cerium is the most reactive element in the lanthanide series due to its electropositive nature. Cerium exists in two different oxidation states (Ce^3+^ and Ce^4+^) or in a mixed valence state. The Ce^4+^ oxidation state is more stable than Ce^3+^. Cerium has two main types of oxides—cerium dioxide (CeO_2_) and cerium sesquioxide (Ce_2_O_3_), nevertheless the more stable CeO_2_ is used rather than Ce_2_O_3_ [24]. This interchangeability of Ce^3+^ and Ce^4+^ makes them regenerative (Fig. 1) [23]. Cerium oxide nanoparticles (C-NPs) have been synthesized using many different methods such as thermal decomposition, sol–gel microemulsion methods, solvothermal oxidation, flame spray pyrolysis and microwave-assisted solvothermal process or mixing cerium sulphate and ammonia solution at room temperature [26]. All C-NPs contain the same core elements (Fig. 2a), but do not display similar biological effects. Prooxidant toxicity of C-NPs was demonstrated in some cases and antioxidant protective effects in others that could be attributed to different physicochemical parameters of the various C-NPs. The method of C-NP synthesis, type of the stabilizing agent used, and the Ce^3+^/Ce^4+^ surface ratio have been reported to play major roles in producing CeO_2_-NPs with different physicochemical properties [23, 26–28]. CeO_2_-NPs mimicking catalase and SOD activities or displaying peroxidase-like activities have been reported [29]. Oxidase-like activity of CNPs originated from surface Ce^3+^ atoms as the catalytic centers. CeO_2_-NPs with lower Ce^3+^ density on the surface show catalase or peroxidase mimetic activity. CeO_2_-NPs can also induce angiogenesis in vivo by modulating the intracellular oxygen environment and stabilizing hypoxia inducing factor 1α (HIF-1α) [30]. CeO_2_-NPs of high surface density of Ce^3+^ inhibit the growth of both gram-negative and gram-positive bacteria [28]. It was reported also that C-NPs can trigger neuronal survival in AD model, beta-amyloid [Aß (25–35)-treated SH-SY5Y cells, twice-subcloned cell line derived from the SK-N-SH neuroblastoma cells], through modulation of the extracellular signal-regulated kinases 1/2 and 5 (ERK1/2, ERK5) and the brain-derived neurotrophic factor (BDNF) signaling pathways [31]. These factors are involved in the signal transduction pathways of neuronal survival and differentiation [32]. There are many reports on the neuroprotective effect of engineered CeO_2_-NPs. Arya et al. [33] reported that CeO_2_-NPs promoted neurogenesis and modulated hypoxia-induced memory impairment through the 5′-adenine monophosphate-activated protein kinase-protein kinase C-cyclic adenosine monophosphate response element-binding protein binding (AMPK-PKC-CBP) protein pathway. Using polyethylene glycol-coated 3 nm nanoceria (CeO_2_-NPs), it was shown that NPs efficiently penetrated the BBB, were localized in rodent brain in adult male Sprague–Dawley rats and decreased OS [32, 34, 35]. Using an in vivo model of mild lateral fluid percussion brain injury in the rat, Bailey et al. [35] demonstrated the beneficial antioxidant action on C-NPs on traumatic brain injury outcome. These authors found that C-NPs improved neural survival as well as cognitive function by decreasing macromolecular free radical damage and promoting preservation of endogenous antioxidant systems [35]. Nanoceria formulation enhances the proliferation and migration of fibroblasts, keratinocytes and vascular endothelial cells, which further accelerate the wound healing process. Nanoceria can also protect regenerating tissue by reducing OS in the wounded region [36]. Nanoceria acted as antioxidants against a free radical-mediated autoimmune degenerative disease in the brain [37]. Nevertheless, cerium cations erode from NPs, which causes strong toxicity to normal tissues and cells in vitro. The increased ROS in cultured human bronchial epithelium, normal (BEAS-2B) cells by C-NPs trigger activation of cytosolic caspase-3 and chromatin condensation, which means that C-NPs can exert cytotoxicity inducing the apoptotic process [38].

Boron cluster-containing redox nanoparticles (B-RNPs) are RNPs, which are synthesized for boron neutron capture therapy. Boron cluster-containing redox nanoparticles are prepared from poly(ethylene glycol)-b-poly((closo-dodecaboranyl)thiomethylstyrene) (PEG-b-PMBSH). This polymer is obtained by the reaction of ^10^B-enriched sodium borocaptate (BSH) with poly(ethylene glycol)-b-poly[4-(2,2,6,6-tetramethylpiperidine-1-oxyl) aminomethylstyrene] (PEG-b-PMNT). B-RNPs are dedicated to cancer therapy, because of their low adverse effects, selective uptake in cancer cells, specific accumulation, long retention in tumour tissue and ROS scavenging ability. After thermal neutron irradiation, cancer cell damage and suppression of tumour growth were observed. Using ^1^H NMR, the presence of both PEG-b-PMBSH and PEG-b-PMNT (nitroxide radicals-containing redox nanoparticle) in the core was confirmed. Figure 2b shows structure of PEG-b-PMBSH [39].

**Fig. 3:**
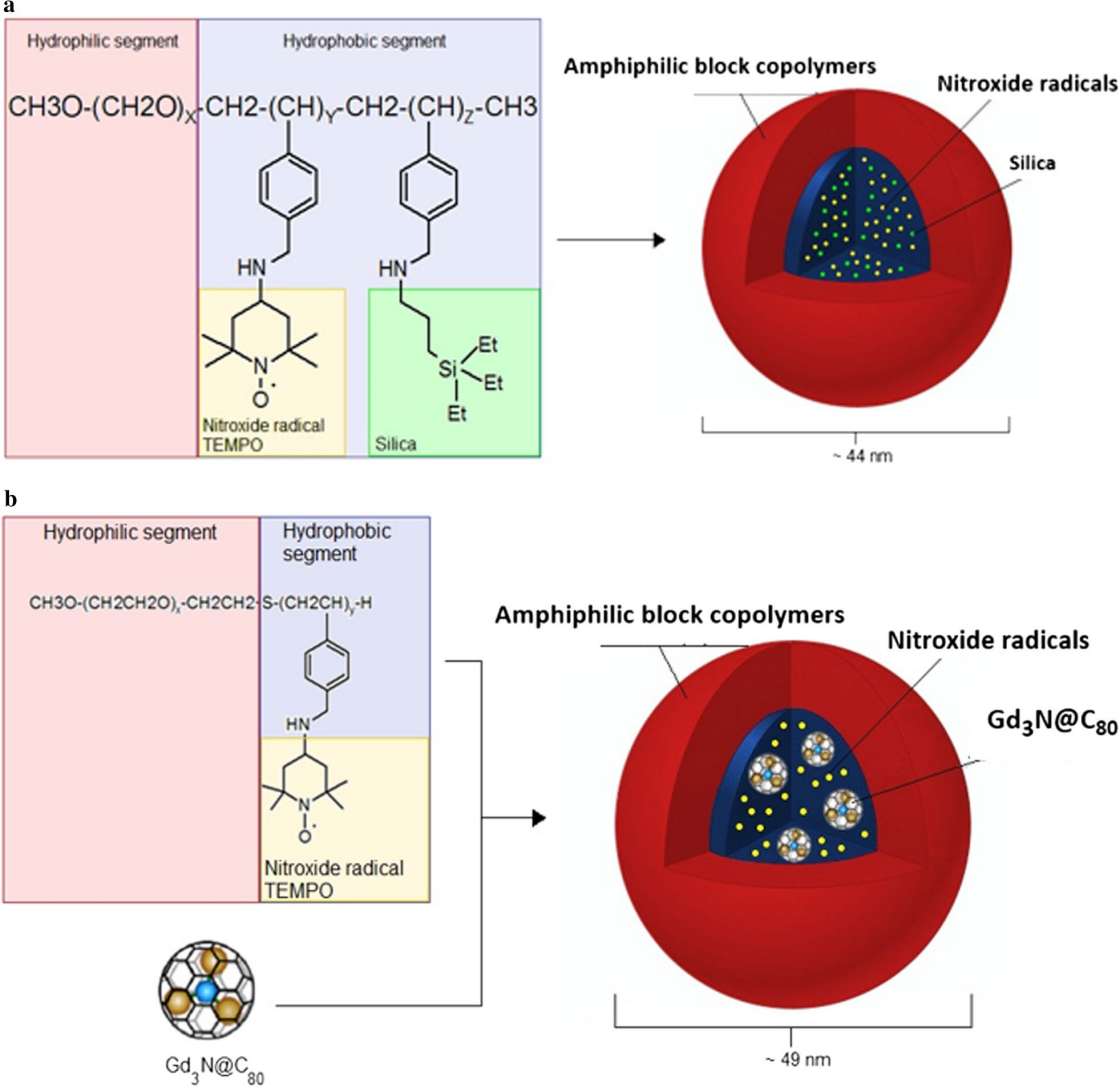
Structure of **a** silica-containing redox nanoparticle (Si-RNP) and **b** Gd_3_N@C_80_ encapsulated redox nanoparticle (Gd_3_NP)

Silica-containing redox nanoparticles (Si-RNPs) are a special type of NPs, which can have medical applications as novel nano-sized adsorbents for peritoneal dialysis, and orally administrable drug carriers for the treatment of gastrointestinal inflammation. These RNPs consist of silica NPs and amphiphilic block copolymers with nitroxide radicals (Fig. 3a). Electrostatic interactions between the cationic segment of a polymer in the core and the entrapped silica nanoparticles form a crosslinked structure that provides siRNP stability in vivo, even under harsh conditions in the gastrointestinal tract. Si-RNPs can be applied not only as adsorbents of body wastes, but also as drug carriers with high loading capacity due to their excellent adsorption properties [40].

Gd_3_N_@_C_80_ encapsulated redox nanoparticles (Gd_3_NPs) is a novel contrast agent for magnetic resonance imaging (MRI). These RNPs contain gadolinium ions in the core. Because free Gd(III) ions are toxic, it is necessary to make Gd-chelate compounds or encapsulate the ions inside RNPs. Gd_3_N_@_C_80_ encapsulated RNP structure (Fig. 3b) is based on typical poly(ethylene glycol) shell with encapsulated fullerene. Inside the hollow sphere structured from 80 carbon atoms, three atoms of Gd are placed. This NP is obtained by reaction of PEG-b-PMNT with Gd_3_N_@_C_80_. Gd_3_N_@_C_80_ particles are characterized by low toxicity, high relaxivity and solubility in water. Gd_3_NPs have the ability to scavenge ROS. In addition, Gd_3_NPs have also colloidal stability, long circulation time and can be used as nano-contrast agent in MRI technique for clinical diagnosis [41].

### Nitroxide radicals-containing redox nanoparticles (NRNPs)

**Fig. 4:**
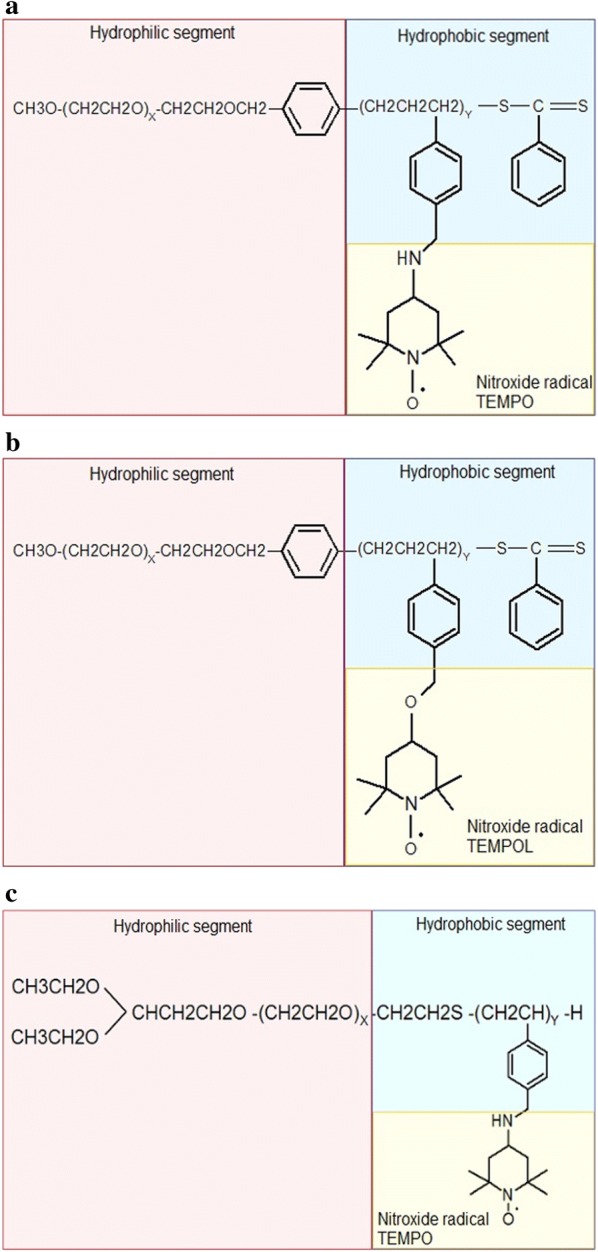
Chemical structure of nitroxide radicals-containing redox nanoparticles (NRNPs): **a** MeO-PEG-b-PMNT, **b** MeO-PEG-b-PMOT and **c** Acetal-PEG-b-PRAS

**Fig. 5:**
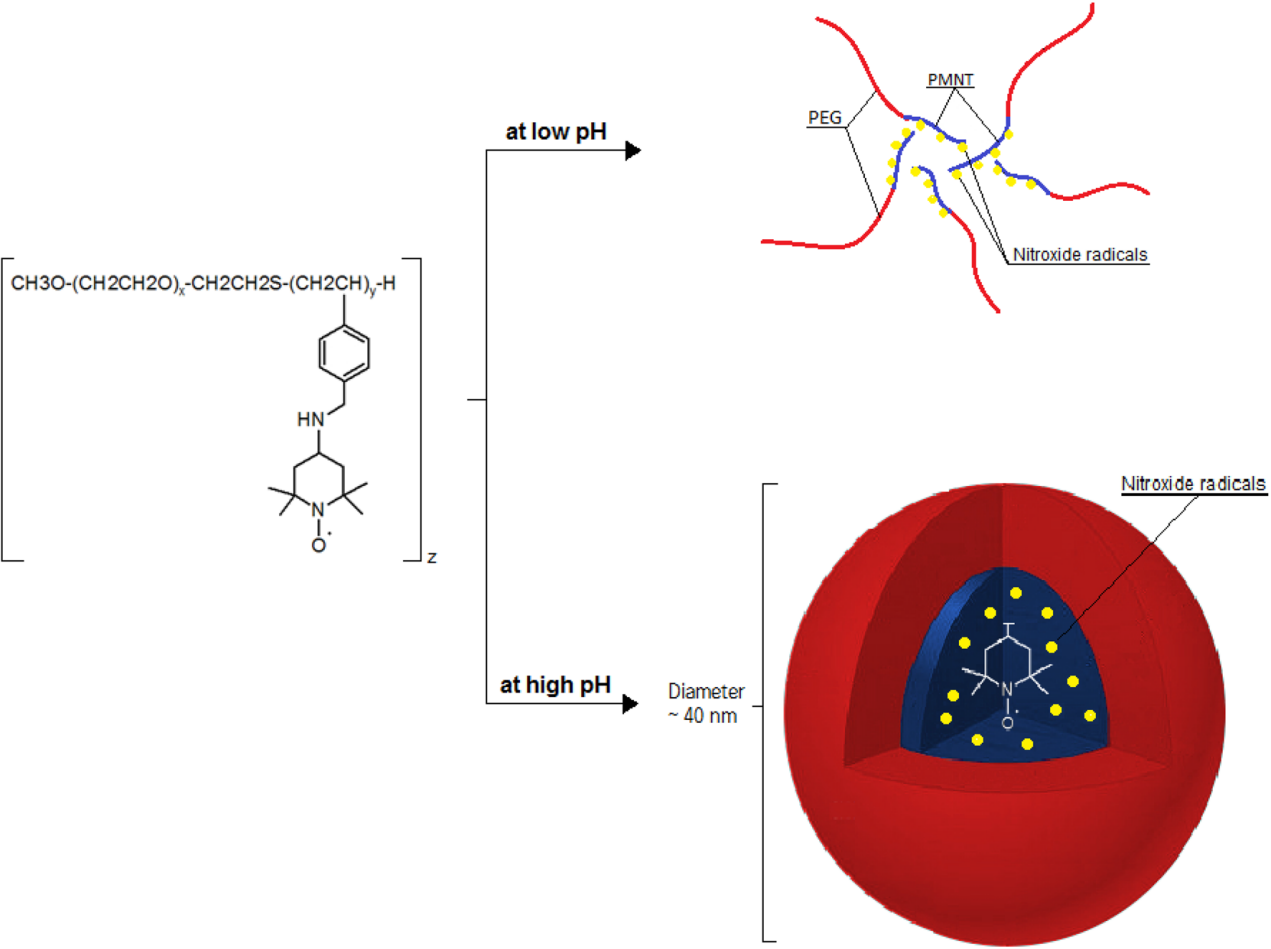
Influence of pH value on the structure of NRNP^N^

Nitroxide radicals-containing redox nanoparticles confine nitroxide radicals to their core. For this reason, their antioxidative nature is not fully expressed when they have the full, spherical form. Nitroxide radicals-containing redox nanoparticles are thought to have the potential to be used as high-performance bio-nanoparticles in vivo [42]. When PEG-b-PCMS is mixed with 4-amino-TEMPO or in the presence of sodium hydride in dimethyl sulfoxide, PEG-b-PMNT is obtained (NRNP^N^; Fig. 4a, c). When 4-hydroxy-TEMPO (TEMPOL) is used instead of 4-amino-TEMPO, poly(ethylene glycol)-b-poly[4(2,2,6,6-tetramethylpiperidine-1-oxyl)oxymethylstyrene] (PEG-b-PMOT) is obtained (NRNP^O^; Fig. 4b). 2,2,6,6-Tetramethylpiperidine-1-oxyl (TEMPO) groups as a side chain of the PMNT segment are known to be stable radicals not reacting with each other. As mentioned before, TEMPO molecules have a pseudo-superoxide dismutase activity; they directly react with both carbon-centered and peroxy radicals preventing the conversion of hydrogen peroxide to hydroxyl radical. Hence, NRNP^N^ may attenuate the formation of hydroxyl radical. Because the PMNT segment possesses repeating amino groups, it protonates in response to pH decrease and becomes hydrophilic to result in disintegration under acidic conditions (pK_a_, 6.5) (Fig. 5). The sensitivity of NRNP^N^s micelles to pH changes is an important considering the low pH extracellular environment of tumour tissues, which could affect treatment efficacy. pH-insensitive redox nanoparticles (NRNP^O^s), which are not disintegrated in any area of the gastrointestinal tract, maintain a micellar form in the stomach and intestine. After oral administration, NRNP^O^s are accumulated on the surface of the intestinal epithelium, but not inside the villi, because diffusion of NRNP^O^s across the intestinal mucus layer to reach the epithelium is difficult, given its size of 40 nm. Contrary to NRNP^O^, the redox polymer after disintegration of NRNP^N^ is internalized deeply in the villi across the intestinal epithelium [21]. A few examples of synthesis methods of selected NRNPs are described below.

### *Poly(ethylene glycol)*-*b*-*poly[4*-*(2,2,6,6*-*tetramethylpiperidine*-*1*-*oxyl)aminomethylstyrene]* (MeO-PEG-*b*-PMNT)

The synthesis of NRNPs is a several-step process. At first, poly(ethylene glycol) monomethyl ether is used to prepare poly(ethylene glycol)-b-chain transfer agent (PEG-b-CTA). In the next step, block copolymer is synthesized by the method of the radical polymerization of chloromethylstyrene (CMS). The product of the reaction is poly(chloromethylstyrene) (PCMS). In result, methoxy-poly(ethylene glycol-b-poly(chloromethylstyrene) is obtained and its main segments are a hydrophilic PEG and a hydrophobic PCMS. To obtain PEG-b-PMNT, chloromethyl groups on the PCMS segment of the block copolymer are converted to nitroxide radicals via amination of MeO-PEG-b-PCMS with 4-amino TEMPO in dimethyl sulfoxide (DMSO). MeO-PEG-b-PCMS is converted to MeO-PEG-b-PMNT (Fig. 4a). The last step relies on preparation of RNP^N^ micelles. MeO-PEG-b-PMNT is used to create micelles using a dialysis method. At first, this polymer is dissolved in dimethylformamide and the solution is dialyzed against distilled water. As a result, NPs with a diameter of 40 nm are formed. The driving force for micelle formation are hydrophobic interactions of NH_2_-TEMPO, which is the active segment.

MeO-PEG-*b*-PMNT, possessing the cationic PMNT segment, may be internalized in the brain via the endocytosis pathway. Interestingly, it can prevent efflux of chemotherapeutic drugs, e.g. doxorubicin, by inhibiting *P*-glycoprotein, the main protein conferring multidrug resistance [43].

Furthermore, the long-term access of redox polymers coupled to serum protein to the brain vessel wall by extended blood circulation time might increase internalization tendency in the brain. Since redox catalytic species are covalently conjugated to redox polymers as stated above, they are internalized together with the polymers. It was reported that 5–6% of the injected in mice dose of the redox polymers (NRNP^O^) was absorbed into the bloodstream and circulated over 24 h while orally administered TEMPOL was eliminated within 1 h from the blood [20].

### *Methoxy-poly(ethylene glycol)-b-poly[4-(2,2,6,6-tetramethylpiperidine-1-oxyl)oxymethylstyrene]* (MeO-PEG-*b*-PMOT)

This NRNP^O^ is a core–shell-type polymeric micelle 40 nm in diameter (Fig. 4b). Methoxy-poly(ethylene glycol)-*b*-poly(chloromethylstyrene) (MeO-PEG-*b*-PCMS) was synthesized by radical telomerization of chloromethylstyrene with methoxy-poly(ethylene glycol)-sulphanyl as a telogen. The polymer backbone of MeO-PEG-*b*-PCMS consists of PEG for the hydrophilic segment and 14 repeating units of PCMS for the hydrophobic segment [20]. Nitroxide radical moieties were introduced as a side chain of the PCMS segment via Williamson ether synthesis of benzyl chloride in the MeO-PEG-b-PCMS block copolymer with the alkoxide of TEMPOL [44].

### *TEMPO-containing acetal-PEG-b-PCMS*

The anionic ring-opening polymerization of ethylene oxide was carried out using potassium 3,3-diethoxypropanolate as an initiator, followed by mesylation with methanesulfonyl chloride to obtain acetal-poly(ethylene glycol)-methanesulfonate (acetal-PEG-Ms) (Compound 1). Compound 1 was reacted with potassium *O*-ethyldithiocarbonate, followed by treatment with *n*-propylamine to obtain heterobifunctional PEG derivatives containing both sulfanyl as well as acetal terminal groups (acetal-PEG-SH) (Compound 2). Poly(ethylene glycol)-block-poly(chloromethylstyrene) (acetal-PEG-b-PCMS) (Compound 3) was synthesized by the free-radical telomerization of CMS using Compound 2 as a telogen. The chloromethyl groups in the PCMS segment of the block copolymer (Compound 3) were quantitatively converted to 2,2,6,6-tetramethylpiperidinyloxyl (TEMPO) groups via the amination of Compound 3 with 4-amino-TEMPO to obtain acetal-PEG-b-PCMS containing TEMPO moieties (Compound 4) (Fig. 4c). The obtained Compound 4 formed core–shell-type NP in aqueous media when subjected to dialysis: the semi-invariant average diameter of the NP was about 40 nm, and NP emitted intense electron paramagnetic resonance (EPR) signals. The TEMPO radicals in the core of NPs were resistant to reduction even by 3.5 mM ascorbic acid. This means that these NPs are high-performance NPs that can be used in vivo [45].

The NRNP showed almost no cytotoxicity up to the 8 mM level, which is in sharp contrast to LMW TEMPO and 4-amino-TEMPO: the median cytotoxic concentrations (IC_50_) of TEMPO and 4-amino-TEMPO were 8.3 and 4.8 mM, respectively (pH < 6). Even at 600 mg/kg mouse body weight (concentration of amino-TEMPO moieties: 960 µmol N[TEMPO]/kg), 60% of IRC mice survived [46].

The compartmentalization of the TEMPO radicals in the NRNP core improves their stability in the bloodstream. Since an amino group was introduced in each repeating unit of the PC-TEMPO segment, the disintegration of the NRNP is caused by protonation of the amino groups in response to the acidic pH environment (pH < 6.0) [46]. The polymer structure is similar for the majority of NRNPs: it is an amphiphilic block polymer with nitroxide radicals inside the core [45, 47–49] (Fig. 4c).

Potential applications NRNPs are based on their several features:

- The disintegration of NRNP^N^ in response to low pH; NRNPs disintegrates after intravenous administration in response to low pH environments, such as ischemic, inflamed and tumor tissues, owing to protonation of amino groups of the hydrophobic segment, resulting in increased ROS scavenging activity, because of an exposure of the nitroxide radicals from the NRNP^N^ core. Orally administered NRNP^N^s are disintegrated to redox polymers in the stomach; redox polymers are absorbed in the blood stream through the intestines and circulate for ca. 24 h, and ultimately reach the liver. Contrary to LMW antioxidants, NRNPs and disintegrated polymers cannot be internalized into normal cells [48]. Additionally, NRNPs suppress liver inflammation and reduced the infiltration of neutrophils, monocytes and macrophages [42].
- RNPs are characterized by long blood-circulation time; The EPR signals in the blood stream are hardly observed even after 2 min, when LMW TEMPOL is administered via tail-vein injection. The half-life of TEMPOL in blood has been reported to be approximately 15 s [50]. On the contrary, when NRNPs prepared from PEG-b-PMNT (NRNP^N^) and PEG-b-PMOT (NRNP^O^) are administered, the ESR signal in the blood stream was observed even 2 h after the tail-vein injection of NRNPs. The half-life of the NRNP^N^s was 60-times longer (15 min) than that of LMW TEMPOL. RNP^O^s show much longer circulation. Actually, the half-life of the NRNP^O^s was 600 min, 2400-times longer than that of LMW TEMPOL [42].
- Low toxicity: The extremely low toxicity of the NRNP^N^ is probably due to the fact that the outer PEG layer constitutes an excellent stealth shield around the amino TEMPO moieties in the NRNP core [42].
- NRNPs are excellent free radical scavengers.
- NRNPs are also potential ESR imaging tool [46]. To visualize, monitor or record spatiotemporal distribution of molecular and cellular processes, imaging modalities such as optical imaging (luminescence and fluorescence), radionucleotide-based imaging [positron emission tomography (PET), single photon emission computed tomography (SPECT)], magnetic resonance imaging (MRI), computed tomography (CT) and ultrasound need specific molecular probes to target molecules, cells or tissues and allow them to be imaged. A desired molecular imaging probe should provide high binding affinity and specificity to target, high sensitivity and high stability in vivo as well as less toxicity. More recently, chlorin e6 (Ce6) and alpha-tocopherol succinate (TOS) were conjugated to LMW heparin via cysteamine as the redox-sensitive linker, forming amphiphilic Ce6-LMWH-TOS (CHT) polymer, which could self-assemble into NPs in water and encapsulate paclitaxel (PTX) inside the inner core (PTX/CHT NPs). The enhanced near-infrared (NIR) fluorescence intensity and ROS generation of Ce6 were observed in a reductive environment, suggesting the cysteamine-switched “ON/OFF” of Ce6. The redox-responsive PTX/CHT NPs successfully integrated the chemotherapeutic effect of PTX, photodynamic therapy and NIR imaging capacities of Ce6, displaying great potential for smart NIR imaging-guided tumor combinational therapy [51]. A summary of properties of various redox nanoparticles is given in Table 1.

**Table 1:** Redox nanoparticles: examples with characteristics

Redox nanoparticle	Abbreviation	Type	Toxicity	Size (nm)	Short characteristics
Poly(ethylene glycol)-block-poly[4-(2,2,6,6-tetramethylpiperidine-1-oxyl) aminomethylstyrene]	PEG-b-PMNT	NRNP^N^	Low—does not show any cytotoxicity up to the concentration of 8 mM	~ 50	Effectively removes ROS at the site of post-reperfusion syndrome Can be used as a new type of drug in redox nanomedicine Alternative treatment for *N*-acetylcysteine (NAC)-side effects like fever, rash, low blood pressure [42]
Methoxy-poly(ethylene glycol)-block-poly[4-(2,2,6,6-tetramethylpiperidine-1-oxyl)aminomethylstyrene	MeO-PEG-b-PMNT	NRNP^N^	No toxicity found	40	ROS-scavenging ability; effective protection of neuronal cells against OS [21]; biocompatible, stable, colloidal; long-term circulation ability in the bloodstream; protection against radiation-induced weight loss; reduction of the ROS-associated skin aging and skin damage caused by UV exposure [52]; potential use for oral treatment of non-alcoholic fatty liver disease; absorption to blood after disintegration in stomach; reduction of inflammation and liver fibrosis [22]; disintegration in acidic environments through protonation of amino groups in the RNP^N^ core after oral administration; inflammation reduction; inhibition the development of colitis-associated colon cancer [53]
Methoxy-poly(ethylene glycol)-block-poly[4-(2,2,6,6-tetramethylpiperidine-1-oxyl)oxymethylstyrene	MeO-PEG-b-PMOT	NRNP^O^	Concentrations of up to the 10 mg/mL do not show any cytotoxicity	~ 40	Long blood circulation ability; ROS-scavenging ability and antioxidant activity; treatment significantly enhanced survival of zebrafish (*Danio rerio*) larvae treated with ROS inducers; induction of the expression of Nrf2 target gene (GSTP1) in the larvae’s intestines and livers; exhibits a potent therapeutic effect and extremely low toxicity to zebrafish embryos [54] so is a promising candidate for clinical trials
Acetal- poly(ethylene glycol)-block-poly(nitroxyl radical-containing aminomethyl-styrene) or TEMPO-containing acetal-poly(ethylene glycol)-block- poly(chloromethylstyrene)	Acetal-PEG-b-PRAS	NRNP^N^	Low—does not show any cytotoxicity up to a concentration of 8 mM	~ 40	High-performance NPs; ROS-scavenging ability [55]
	TEMPO-containing acetal-PEG-b-PCMS				
Silica-containing RNP	Si-RNP	NRNP^N^	No toxicity found	43.6–55.4	Stability at low pH; slower drug releasing and high accumulation in the colon; easier adsorption of drug on silica; more effective drug loading; anticancer activity [19]; ROS-scavenging ability [40]
Gd_3_N@C_80_ encapsulated RNP	Gd_3_NP	NRNP^N^	No toxicity found	~ 50	ROS-scavenging ability; colloidal stability; long-term circulation ability Nano-contrast agent for MRI for clinical diagnosis [41]
Boron cluster-containing RNP	B-RNP	NRNP^N^	No toxicity found	36	ROS-scavenging ability; high colloidal stability; ability to damage tumor cells; ability to suppress the tumor growth and metastasis; huge potential in Boron Neutron Capture Therapy as a boron delivery system [39]

### Redox nanoparticles as a novel treatment approach for treatment of neurodegenerative diseases

AD is a primary degenerative disease of the CNS, characterized by progressive deficit of memory and other cognitive functions, such as the learning ability, thinking, problem-solving and language communication. In 2015, there were approximately 48 million people worldwide with AD and the abundance of this disease has a growing tendency due to the increase in the lifespan in the developed countries. Alzheimer’s disease is one of the most financially costly diseases [56]. The most characteristic hallmarks for this disease during a neuropathological examination of the brain are β-amyloid (Aβ) plaques and hyperphosphorylated τ (tau) deposits and fibrillar degeneration [57]. β-amyloid acts as a prion-like protein, which means that Aβ seeds can be intracellular oligomers. More recently, Olsson et al. [58] established that intracellular inclusions of Aβ can be seeded and stably maintained in an APP expressing cell line. These inclusions can propagate both vertically (stable phenotype over time) and horizontally [inclusion can be induced in naive N2a cells expressing human amyloid precursor protein (APP) with the Swedish mutation (APPswe)] [58]. Parkinson’s disease (PD) affects about 1–2% of the population aged over 65 and about 4% of the population aged over 85. However, some people develop the disease at younger age. Some doctors consider anyone diagnosed PD under the age of 50 to have young-onset PD (YOPD), while others set the age limit of 40 [59]. Primary motor signs of PD include tremor of the hands, arms, legs, jaw and face, bradykinesia, rigidity or stiffness of the limbs and trunk, impaired balance and coordination. PD is characterized by progressive degeneration and decay of dopaminergic neurons of the nigrostriatal path and, to a lesser extent, of the mesocorticolimbic and hippocampal path and, with variable intensity, noradrenergic, serotoninergic as well as cholinergic neurons, due to the deposition of insoluble protein aggregates, such as α-synuclein (αSyn) and parkin, forming so-called Lewy’s bodies in the cytoplasm [60]. Till now, there is no cure for AD and PD, with the currently recommended treatments causing only transient alleviation of symptoms of these diseases. The need for new medicines is enormous, taking into account the socioeconomic dimension of these neurodegenerative diseases. Evidence for the involvement of OS and NS in the progress of AD and PD is compelling. The brains of patients suffering AD present a significant extent of oxidative damage associated with the abnormal marked accumulation of Aβ and the deposition of neurofibrillary tangles. Metals such as iron copper and zinc seem to play an important role in the induction of OS [61]. There are high affinity binding sites for copper and zinc on the N-terminal, metal-binding domains of Aβ and its APP. Copper is a potent mediator of the highly reactive hydroxyl radical (OH·) formation, and consequently contributes significantly to the generation of OS characteristic of AD brain. High concentrations of copper have repeatedly been found in amyloid plaques [62]. In addition, high concentrations of zinc were associated with memory and cognitive regions of the brain, including the neocortex, amygdala and hippocampus, which are mostly affected in AD pathology. The binding of zinc induces a highly ordered conformational state of Aβ (1–40), leading to the production of toxic fibrillary Aβ aggregates. Moreover, the immunological/inflammatory response to insoluble Aβ plaques involves the disruption of zinc homeostasis followed by uncontrolled cerebral zinc release, which is typical for OS. Thus, the uncontrolled accumulation of zinc and Aβ leads to zinc-induced and Aβ-mediated OS and cytotoxicity [63]. Brain cell membrane phospholipids are characterized by a high content of polyunsaturated fatty acids, which are particularly vulnerable to free radical attacks. Increased lipid peroxidation is one of the most prominent features in the AD brain [64]. Proteins, which are the most abundant organic compounds in the cells, are the main target of ROS and RNS. Protein oxidation is markedly increased in AD. The oxidation of brain proteins can affect enzymes critical to neuron and glial functions. This is the case for two enzymes especially sensitive to oxidative modification, glutamine synthetase and creatine kinase, which are markedly reduced in AD brains [65]. Inactivation of glutamine synthetase results in alterations of glutamate concentrations and enhancement of excitotoxicity, whereas oxidative impairment of creatine kinase may decrease energy metabolism [66]. The pathologic aggregation of protein leads to fibril formation and insolubility. Neurofibrillary tangles are characterized by the aggregation and hyperphosphorylation of the τ protein into paired helical filaments. Phosphorylation is linked to oxidation through the microtubule-associated protein kinase pathway and through activation of the transcription factor nuclear factor-κB, thus potentially linking oxidation to the hyperphosphorylation of the τ protein. Protein oxidation is also capable to facilitate advanced glycation end products as a posttranslational modification of proteins. Furthermore, OS in the brain affects DNA, producing oxidation of guanine and other DNA bases, strand breaks, sister chromatid exchange, and DNA–protein crosslinking. Hence, the overproduction of ROS may have a deleterious effect and can be an important mediator of damage to cell structures and consequently significantly contribute to the progress of AD [2]. In the brain of PD patients, elevated iron levels are also observed. The effects of the disease are most marked in the nigrostriatal dopaminergic system, which can be explained by formation of potent redox couple formed by iron and dopamine itself [67]. Copper dyshomeostasis has also been demonstrated in the PD brain [68]. Reduced activity of Complex I of the respiratory chain in the *substantia nigra pars compacta* neurons of patients with PD has been demonstrated; it may contribute to the generation of excessive ROS. Mitochondrial Complex I deficiency in the frontal cortex, fibroblasts, and blood platelets have also been reported in patients with PD. Furthermore, the relationship between mitochondrial dysfunction and PD is supported by the findings that the Complex I inhibitors, such as 1-methyl-4-phenyl-1,2,3,4-tetrahydropyridine (MPTP). 1-methyl-4-phenyl-1,2,3,4-tetrahydropyridine metabolite, 1-methyl-4-phenylpyridinium (MPP^+^), may exert cytotoxic effects on the dopamine neurons, resulting in clinically parkinsonian phenotype, and induce nigral degeneration with cytoplasmic α-Syn [69]. It has been also reported that genetic mutations in proteins including α-Syn, parkin, phosphatase and tensin homolog-induced putative kinase (PINK) were linked to the familial forms of PD. Mutations of these genes have been known to affect mitochondrial function and increase OS [70]. Changes in the levels of antioxidant molecules have also been reported even in the early stage of PD. For example, the levels of glutathione, a major cellular antioxidant, have been reduced in the *substantia nigra pars compacta* in PD [71]. It has been suggested repeatedly that both AD and PD patients can benefit from antioxidant supplementation and, since transition metals are important factors in the generation of OS in these diseases, from treatment with metal chelators. However, metal chelation may deplete the body of metals essential for the vital functions of the organism. The problems with BBB penetration by many chelators can make them active mainly outside the brain. The use of nitroxides may be a solution to both these needs. Unlike most antioxidants, which reduce metal ions facilitating their participation in the Fenton reaction, nitroxides are efficient antioxidants, but they oxidize transition metal ions preventing them from being involved in the Fenton reaction [8]. In view of the existence of common features between AD and diabetes [72] the anti-glycating activity of nitroxides may be also of value [11]. Free nitroxides are short-lived in vivo and part of them may have difficulties in penetrating the BBB. Covalent linking of nitroxides to nanoparticles able to penetrate BBB can prolong their lifespan and enable them to reach the brain. Appropriate design may create nitroxides combining the antioxidant and metal-oxidizing properties with another therapeutically useful functionality, e.g. inhibition of protein aggregation.

### Neuroprotective role of NRNPs against AD—in vitro studies

Overproduced ROS can be stopped thanks to ROS-scavenging properties of RNPs [6]. Chonpathompikunlert et al. [73] examined the protective effect of NRNPs prepared from MeO-poly(ethylene glycol) (PEG)-b-PCTEMP using human neuroblastoma SH-SY5Y cells treated overnight with different concentrations of NRNPs (0.1–1 mM) prior to exposure to Aβ_1–42_ (20 µM) for 48 h [73]. Thrice-cloned neuroblastoma is one of the few human malignancies known to demonstrate spontaneous regression from an undifferentiated state to a completely benign cellular appearance. SH-SY5Y cells are characterized by a stable karyotype consisting of 47 chromosomes. Different subtypes of neurons can be obtained as a result of the differentiation induced by the chemical compounds [1, 2]. The 24-h pretreatments with free TEMPO (0.5 mM) and NRNPs exhibited a highly protective effect on SH-SY5Y cells against the cytotoxicity of Aβ_1–42_. The effect of NRNPs was much higher than that of free TEMPO. Aβ_1–42_-treated SH-SY5Y cells show an increase in the levels of all products of ROS reactions including lipid peroxidation and protein carbonyl and DNA oxidation products, whereas NRNPs can decrease the levels of all products of ROS [73]. Nagasaki group (2011) also have confirmed that NRNPs (1 mM) prepared from MeO-PEG-b-PCTEMPO (MeO-PEG-b-PMNT) coupled with piperine (1-piperoylpiperidine, PI) (20, 50, 100 µM) show an anti-apoptotic effect on Aβ-induced (20 µM) human neuroblastoma SH-SY5Y cell death in vitro. Piperine (PI) is an alkaloid presents in the fruits of black pepper (*Piper nigrum*) and other piper species (family: *Piperaceae*). It was reported that, the NRNP/PI reduced the ROS level and ROS products compared with those of cells treated with NRNPs alone. The NRNP/PI treatment enhanced catalase and glutathione peroxidase activities [74]. Although most of the previous studies have reported that PI is highly toxic at high concentrations [75–77] no cytotoxicity was observed up to the PI concentration of 100 mM, when the PI was coupled with 1 mM NRNPs. The compartmentalization of piperine in the core of the NRNPs might play an important role in the reduction of the toxic effect of PI.

### In vivo studies of neuroprotective effects of RNPs in neurodegenerative diseases

**Fig. 6:**
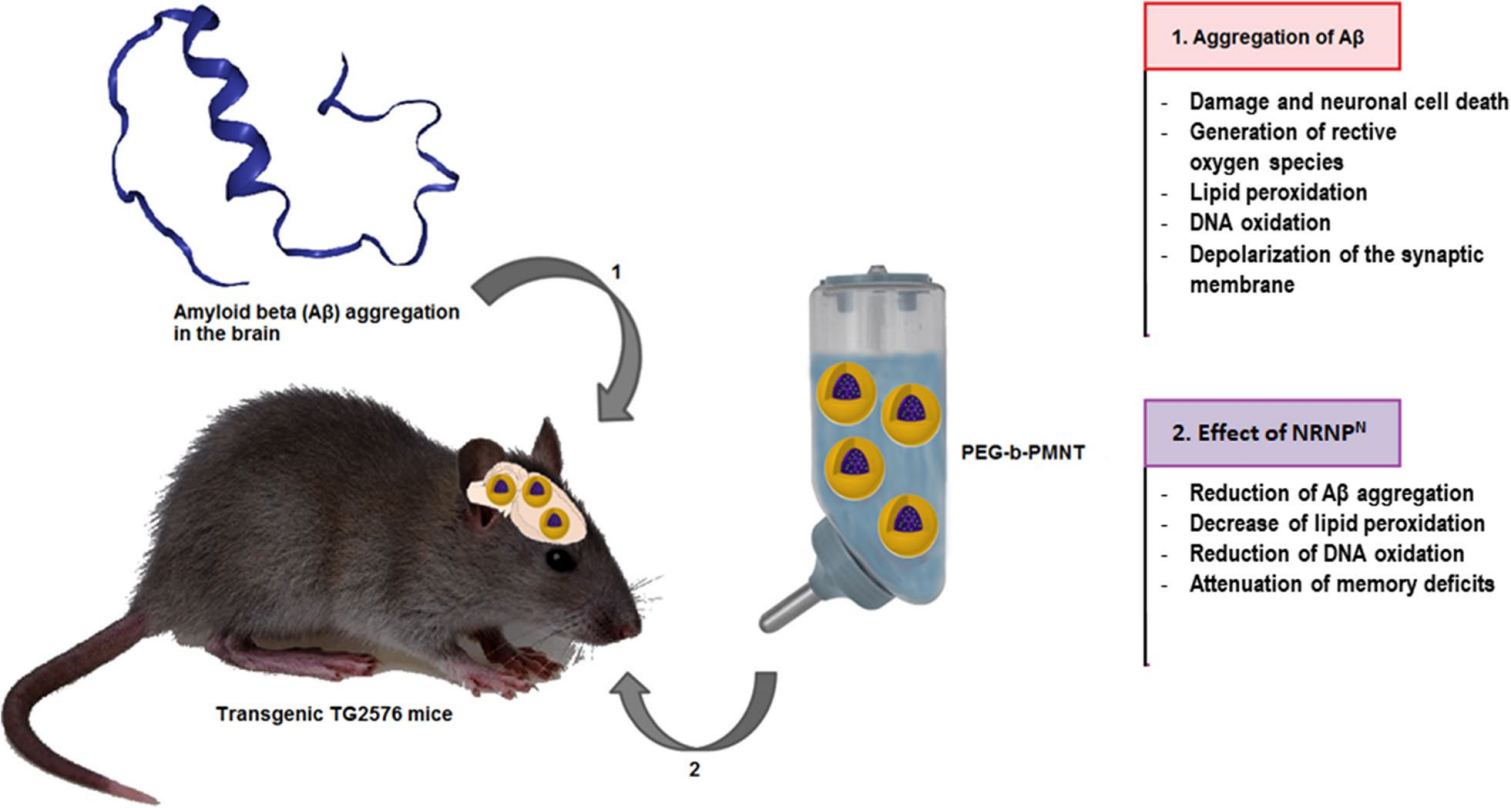
Effect of redox nanoparticles in vivo

The employment of antioxidants in neurodegenerative diseases has been proposed to ameliorate OS/NS. Nevertheless, the effects of antioxidants, among them natural components of the diet are limited and new, more efficient antioxidants are searched for. Vitamin E, LMW antioxidant, was reported to show slight efficacy such as slowing of functional decline of AD in clinical trials although complete recovery was not observed [78]. More recently, drug delivery systems using nanomedicines have attracted much attention in various areas of therapy. Nevertheless, most nanomedicines are not used as orally administered drugs for chronic diseases, such as AD and PD, because nanoparticles between the sizes of 10 and 100 nm are not absorbed via the gastrointestinal tract, as discussed before [21, 55]. An orally administered drug with nanomedicine-like characteristics absorbed in the blood can be an ideal oral medication for such systemic diseases. Nagasaki group (2011, 2012) have proposed “*redox polymer nanotherapeutics*” using amphiphilic block copolymer, poly(ethylene glycol)-*b*-poly[4-(2,2,6,6-tetramethylpiperidine-1-oxyl)aminomethylstyrene] (MeO-PEG-b-PMNT) (10 kDa, referred to as a redox polymer) [42, 79]. This redox polymer possesses nitroxide radicals (NRNP^N^) in bound covalently the hydrophobic segments and forms a polymeric micelle under physiological conditions, which confines the nitroxide radicals in its core and is 40 nm in diameter. As discussed above, NRNP^N^ disintegrates after intravenous administration in response to low pH environments, such as inflamed, ischemic as well as tumor tissues, resulting in increased ROS scavenging activity [48]. Until now, the therapeutic effect of intravenously administered NRNP^N^ was confirmed for various OS/NS-induced injuries including intracerebral hemorrhage and ischemia–reperfusion injuries of the kidney, heart and brain) [21, 82]. The administration of TEMPO in NRNP exerted cardioprotective effects against ischemia and reperfusion injury in canine hearts without exerting unfavourable hemodynamic effects [80]. Moreover, NRNP^N^ do not cause adverse effects, because of no internalization in healthy cells such as blood cells and colon mucosal cells [20, 53]. Nagasaki group [53] reported that orally administered pH-sensitive NRNP^N^ (prepared by the self-assembly of MeO-PEG-*b*-PMNT (MW [PEG] = 5500 Da; MW [PMNT] = 4500 Da) using the dialysis method revived the cognition in 17-week-old the senescence accelerated mouse (SAMP8) mice [21], which are a spontaneous animal model of overproduction of APP and oxidative damage [81]. Small, but evident, amounts of NRNP^N^ were internalized in the brain of normal mice. After treatment with NRNP^N^, ROS levels were decreased significantly in the brain of SAMP8 mice, probably because of the long access of redox polymers to blood vessel in brain. The scavenging of ROS in the brain prevented OS and resulted in recovery of endogenous antioxidant enzyme activities, thus protecting neuronal cells effectively. Furthermore, orally administered NRNP^N^ did not show any detectable toxicity to main organs [21]. More recently, Hosoo et al. [82] reported that an intra-arterial injection of a novel NRNP^N^, containing 4-amino-TEMPO in the core, after cerebral ischemia–reperfusion injury reduced BBB damage and infarction volume by improving multiple ROS scavenging capacities. Therefore, NRNPs can provide neurovascular unit protection [82]. Boonruamkaew et al. [83] used the polymer PEG-b-PMNT as a neuroprotector. The PEG segment is the water-soluble part, while PMNT is the water-insoluble part. The TEMPO groups catalytically react with ROS. These authors confirmed that oral administration of NRNP^N^ prevents against Aβ accumulation in Tg2576 mice overexpressing a mutant form of APP. It was shown that NRNP^N^-treated mice had significantly attenuated cognitive deficits of both spatial and non-spatial memories. NRNP^N^ treatment increased inhibition of superoxide dismutase and glutathione peroxidase activity, neuronal densities in the cortex and hippocampus, decreased Aβ(1–40), Aβ(1–42) and gamma (γ)-secretase levels, and reduced Aβ plaque [83]. In summary, RNP^N^ may be a promising candidate for AD therapy because of its antioxidant properties and reduction in Aβ aggregation, thereby suppressing its adverse side effects (Fig. 6).

### Scope of future applications

Neurodegenerative diseases, like AD and PD, are a group of disorders that have an increasingly high prevalence along with the shortage of effective treatments. Based on results of current studies, RNPs may be useful in the therapy of various diseases. Because of their nano-size and surface hydrophobicity, they are capable of penetrating some cells (e.g. cancer cells) and crossing BBB. Their antioxidant properties can help to decrease cell damage caused by free radicals. These particles are biocompatible; moreover, NRNPs have long-term blood circulation and low toxicity. Their surface properties can be modified to obtain better targeting. All these unique properties make RNPs very promising for the treatment for neurodegenerative diseases and their application in animal models and clinical studies may be expected.

## Conclusion

Neurodegenerative diseases, like AD and PD, are a group of disorders that have in common their increasingly high prevalence along with the shortage of effective treatments. The use of nanomedicine, nanoscale structures for drug delivery, exhibits a really high therapeutic potential in the field of neurodegenerative diseases therapy. Based on results of current studies, redox nanoparticles have many potential applications in nanomedicine. They could be used especially as drug delivery systems. Because of their nano-size and surface hydrophobicity, they are capable of penetrating altered cells (e.g. cancer cells) and crossing BBB. Their antioxidant properties can help remove ROS and thus decrease cell damage caused by free radicals. These anti-inflammatory and potentially anti-ageing particles are biocompatible; moreover, RNPs have long-term blood circulation and low toxicity. They also could protect against ionizing radiation. All these unique properties make RNPs the foundation of innovative methods in treatments for neurodegenerative diseases.

## Abbreviations

Aβ: β-amyloid
AD: Alzheimer’s disease
APP: amyloid precursor protein
αSyn: α-synuclein
BBB: blood–brain barrier
BDNF: brain-derived neurotrophic factor
B-RNPs: boron cluster-containing redox nanoparticles
BSH: ^10^B-enriched sodium borocaptate
CMS: chloromethylstyrene
C-NPs: cerium oxide nanoparticles
CNS: central nervous system
DHR: dihydrorhodamine
EO: ethylene oxide
ESR: electron spin resonance
HSA: human serum albumin
IUPAC: International Union of Pure and Applied Chemistry
LMW: low molecular weight
MeO-PEG-b-PCMS: methoxy-poly(ethylene glycol-b-poly(chloromethylstyrene)
MeO-PEG-b-PMNT: methoxy-poly(ethylene glycol)-b-poly[4-(2,2,6,6-tetramethylpiperidine-1-oxyl)aminomethylstyrene]
MeO-PEG-b-PMOT: methoxy-poly(ethylene glycol)-b-poly[4-(2,2,6,6-tetramethylpiperidine-1-oxyl)oxymethylstyrene]
MPP^+^: 1-methyl-4-phenylpyridinium
MPTP: 1-methyl-4-phenyl-1,2,3,4-tetrahydropyridine
MRI: magnetic resonance imaging
NMR: nuclear magnetic resonance
NP: nanoparticle
NRNPs: nitroxide radicals-containing redox nanoparticles
NRNP^N^: 4-amino-TEMPO-containing redox nanoparticle (nitroxide radical-containing nanoparticles prepared by PEG-b-PMNT)
NRNP^O^: 4-hydroxy-TEMPO-containing redox nanoparticle (nitroxide radical-containing nanoparticles prepared by PEG-b-PMOT)
NS: nitrative stress
OS: oxidative stress
PEG-b-PMBSH: poly(ethylene glycol)-b-poly((closo-dodecaboranyl)thiomethylstyrene)
PEG-b-PMNT: poly(ethylene glycol)-b-poly[4-(2,2,6,6-tetramethylpiperidine-1-oxyl)aminomethylstyrene]
PEG-b-PMOT: poly(ethylene glycol)-b-poly[4-(2,2,6,6-tetramethylpiperidine-1-yl)oxyl oxymethylstyrene]
PD: Parkinson’s disease
PMNT: poly[4-(2,2,6,6-tetramethylpiperidine-1-oxyl)aminomethylstyrene
PROXYL: 2,2,5,5-tetramethyl-1-pyrrolidinyloxyl
RNP: redox nanoparticle
ROS: reactive oxygen species
RNS: reactive nitrogen species
SIN-1: 3-morpholino-sydnonimine
Si-RNP: silica-containing redox nanoparticle
SOD: superoxide dismutase
TEMPO: 2,2,6,6-tetramethylpiperidin-1-yl-oxyl
